# Detection of Methylated SEPT9 in Plasma Is a Reliable Screening Method for Both Left- and Right-Sided Colon Cancers

**DOI:** 10.1371/journal.pone.0046000

**Published:** 2012-09-25

**Authors:** Kinga Tóth, Ferenc Sipos, Alexandra Kalmár, Árpád V. Patai, Barnabás Wichmann, Robert Stoehr, Henriette Golcher, Vera Schellerer, Zsolt Tulassay, Béla Molnár

**Affiliations:** 1 2nd Department of Internal Medicine, Semmelweis University, Budapest, Hungary; 2 Molecular Medicine Research Unit, Hungarian Academy of Sciences, Budapest, Hungary; 3 Institute for Pathology, Universitätsklinikum Erlangen, Erlagen, Germany; 4 Department of Surgery, Universitätsklinikum Erlangen, Erlagen, Germany; Sun Yat-sen University Cancer Center, China

## Abstract

**Background:**

Methylated Septin 9 (SEPT9) is a sensitive biomarker for colorectal cancer (CRC) from peripheral blood. However, its relationship to cancer localization, guaiac-based fecal occult blood test (gFOBT) and carcinoembryonic antigen (CEA) have not been described.

**Methodology/Principal Findings:**

Plasma samples were collected for SEPT9 analysis from patients with no evidence of disease (NED) (n = 92) before colonoscopy and CRC (n = 92) before surgical treatment. DNA was isolated and bisulfite-converted using Epi proColon kit 2.0. Qualitative determination was performed using Epi proColon 2.0 RT-PCR assay. Samples for gFOBT and CEA analysis were collected from NED (n = 17 and 27, respectively) and CRC (n = 22 and 27, respectively). SEPT9 test was positive in 15.2% (14/92) of NED and 95.6% (88/92) of CRC, including 100% (67/67) from stage II to stage IV CRC and 84% (21/25) of stage I CRC when a sample was called positive if 1 out of 3 PCR replicates was positive. In a second analysis (2 out of 3 PCR replicates) specificity improved to 99% (91/92) of NEDs, at a sensitivity of 79.3% (73/92) of SEPT9 positives in CRC. gFOBT was positive in 29.4% (5/17) of NED and 68.2% (15/22) of CRC and elevated CEA levels were detected in 14.8% (4/27) of NED and 51.8% (14/27) of CRC. Both SEPT9 (84.8%) and CEA (85.2%) showed higher specificity than gFOBT (70.6%). SEPT9 was positive in 96.4% (54/56) of left-sided colon cancer (LSCC) cases and 94.4% (34/36) of right-sided colon cancer (RSCC) cases. gFOBT was positive in 83.3% (10/12) of cases with LSCC and 50% (5/10) of cases with RSCC, elevated CEA was detected 60% (9/15) of LSCC and 41.7% (5/12) of RSCC.

**Conclusions/Significance:**

The high degree of sensitivity and specificity of SEPT9 in plasma makes it a better method to detect CRC than gFOBT and CEA, even for the more difficult to detect RSCC.

## Introduction

Colorectal cancer (CRC) is the third most common malignancy worldwide, with more than 1.2 million new cases and 808,700 deaths in the year 2008 [1]. The incidence rates are highest in Europe, Australia, New Zealand, and North America and CRC affects significantly more males than females. If it is detected at an early stage, CRC is curable in most cases.

All of the currently used screening methods, such as fecal occult blood test, CT colonography, flexible sigmoidoscopy, and colonoscopy have limitations [2]. The sensitive fecal occult blood test is a non-invasive and low cost method with limited sensitivity for CRC [2] and only reduces the relative risk of CRC mortality by 15–25% [3]. The standard guaiac-based fecal occult blood test (gFOBT) detects only 33–75% of CRC [2]. The more expensive human hemoglobin-specific fecal immunochemical test (FIT) detects CRC with a sensitivity of about 60–85% [4]. CT colonography is less invasive than colonoscopy and has a sensitivity for detecting CRC or adenomas 10 mm or more in diameter of about 90%. The disadvantages of CT colonography are the need for bowel preparation, special expertise, the use of X-ray and higher cost [5]. In a multicenter randomized controlled study, sigmoidoscopy reduced CRC incidence by 23% and mortality by 31% and reduced the incidence of distal colon cancer (rectum and sigmoid colon) by 50% [6]. The limitations of this diagnostic method are the need for bowel preparation, the relatively high cost, and the inability to detect proximal colonic lesions. The “gold-standard method” of colonoscopy has more than 95% specificity for CRC. Case-control studies have shown reduction of CRC incidence by 53–72% and reduction of mortality by 31% [7], [8]. Although it has the highest specificity, there are several limitations, such as the need for bowel preparation, expertise, cost, invasiveness, availability, low adherence rate, and occasional serious complications. Since the conventional methods for CRC screening are either ineffective or invasive, a more patient-friendly and successive method is needed.

While several minimally invasive, serum-based tumor markers for CRC are available, their specificity and sensitivity are not sufficient. Carcinoembryonic antigen (CEA) has been shown to have a sensitivity of 43.9% at 95% specificity [9]. Chen *et al.* found a sensitivity of 40.9% and specificity of 86.6% for CEA in CRC and when combined with survivin antibodies the sensitivity rises to 51.3% and the specificity to 89.9% [10]. Even so, CEA is not recommended for screening, but it can be used for monitoring response to surgical or systemic therapy [11].

Circulating DNA is found in human peripheral blood serum at increased concentrations in several diseases, such as rheumatic [12] or neoplastic disorders [13]. Methylated DNA has also been found in human serum and Lofton-Day *et al.* tested three such markers in human plasma samples from healthy controls and patients with CRC [14]. Out of these candidates, Septin 9 (SEPT9) proved to be the most specific. Subsequently, a new blood-based colorectal cancer-specific test, the methylated Septin 9 (SEPT9) test, was developed. Several case-control studies have been performed to validate the SEPT9 biomarker [15]–[18] and based on these findings; SEPT9 is an appropriate, minimally invasive biomarker for colorectal cancer. Warren *et al*. detected the SEPT9 methylation according to clinical stage, tumor location and histologic grade of colon cancers using a modified protocol [19].

It remains to be determined whether SEPT9 is a reliable screening method for both left- and right-sided colon tumors. Flat-type neoplasias are more common in the right side of the colon while polypoid-type lesions are more common in the left side. In addition, right-sided colon cancer is more likely to be detected at an advanced stage [20]. Both anatomical and genetic factors result in different specificity and sensitivity according to the localization (left or right side) of the colon tumor. Therefore, the development of a minimally invasive colorectal cancer-specific screening test with sensitivity independent of tumor location would be of great clinical importance.

In this study we analyzed SEPT9 sensitivity and specificity for both left- and right-sided colorectal cancer. In addition, we also compared SEPT9 to a routine fecal-based screening method (gFOBT) and a blood-based tumor marker (CEA) since no such study had yet been performed.

## Materials and Methods

### Ethics Statement

The study was approved by the local ethics committee and government authorities. Written informed consent was obtained from all patients. Detailed interviews for medical history and physical examinations were performed. (Regional and Institutional Committee of Science and Research Ethics, TUKEB Nr: 116/2008).

### Study Design, Patients, and Lower Gastrointestinal Endoscopy

**Figure 1:**
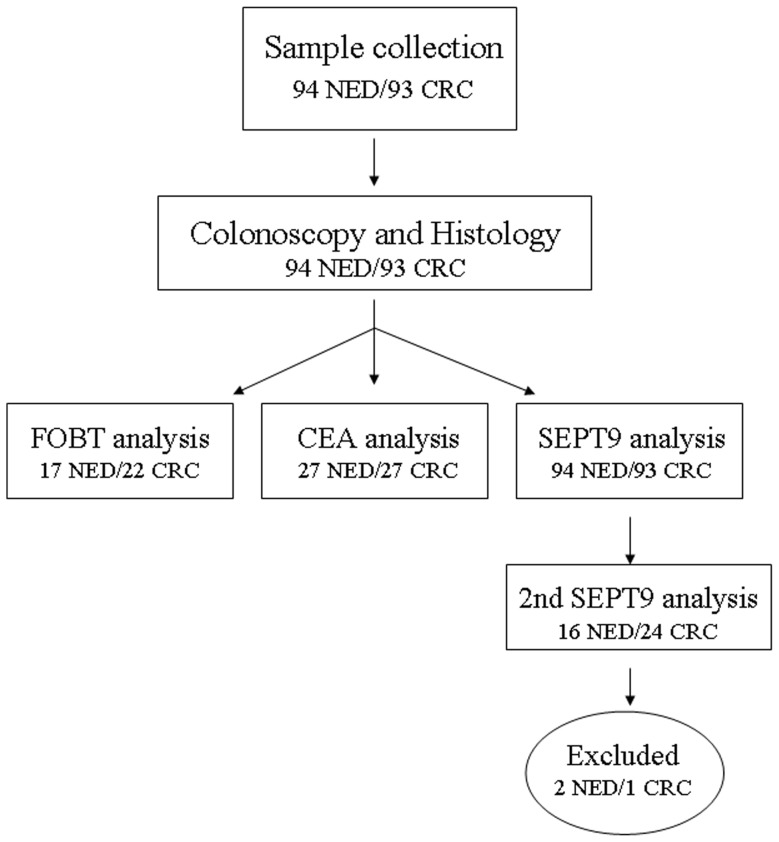
Study design and sample number for each step of the assay. 94 NED and 93 CRC plasma samples were collected. Forty patient samples (16 NED and 24 CRC) were measured twice for SEPT9 validation purposes. 2 NED and 1 CRC samples yielded both Septin 9 positive and negative results; hence they were excluded from the study. Furthermore samples for FOBT and CEA were collected retrospectively. CRC = colorectal cancer; NED = no evidence of disease (healthy control); SEPT9 = Septin 9.

A total of 93 patients with colorectal cancer (CRC) and 94 healthy controls (no evidence of disease; NED) were included in the study. Exclusion criteria were the following: systemic inflammatory, malabsorptive diseases, acute medical conditions, and other malignant diseases. See Table 1 and Table S1 for detailed demographic data. CRC patients were divided into two groups depending on the localization of the cancer in relation to the splenic flexure of the colon: left-sided (n = 36) and right-sided CRC (n = 57). All of the subjects (healthy controls and patients with colorectal cancer) underwent lower endoscopy, during which biopsies were taken for histological examination. In the case of CRC, the patients were stratified by the anatomic appearance of the tumor and then characterized by histopathology. None of the patients with cancer received chemotherapy, radiotherapy, or surgical intervention before endoscopy. The endoscopy in all cases was performed using a videocolonoscope (CF-Q160, Olympus, Hamburg). Peripheral blood samples were taken before colonoscopy using 9.5 ml EDTA tubes (Vacutainer, Becton Dickinson, New Jersey, USA). For validation purposes, 2×9.5 ml peripheral blood samples were taken from 40 patients (16 NED and 24 CRC). Plasma preparation was done from all of the peripheral blood samples by repeated centrifugation for 12 min at 1,350 rcf and plasma samples were stored at −80°C until needed. See Figure 1 for study design.

**Table 1:** Demographic data of patients.

	Healthy controln = 94	Total cancern = 93	Stage I CRCn = 25	Stage II CRCn = 14	Stage III CRCn = 36	Stage IV CRCn = 18
**Gender** **(female/male)**	58/36	45/48	10/15	7/7	19/17	9/9
**Age** **(mean±SD)**	62.6±9.9	67.8±9.8	65.9±9.5	66.4±7.2	69.4±10.0	68.3±11.9

CRC = colorectal cancer.

### DNA Extraction and Qualitative PCR Analysis of SEPT9

DNA extraction from plasma samples and bisulfite conversion were performed using Epi proColon 2.0 test (Epigenomics AG, Berlin, Germany) according to the manufacturer’s instructions. Bisulfite conversion results in the deamination of unmethylated cytosine nucleotides that are eventually converted to uracil nucleotides. Upon PCR amplification, the unmethylated cytosines are replaced with thymine nucleotides while the methylated cytosines remain as cytosines. The subsequent real-time PCR detected the methylated CpG sequences within the v2 transcript of the Septin 9 gene and the total bisulfite-converted DNA of a region of the beta actin gene (ACTB). A methylated Septin 9-specific fluorescent detection probe, bisulfite-converted unmethylated sequence specific blocker and primers designed in regions lacking CpG dinucleotides were used for PCR reactions. Each sample was tested in triplicate during PCR analysis with the LightCycler 480 (Roche Diagnostics, Basel, Switzerland) instrument. In all independent runs, Epi proColon Negative Control and Epi proColon Positive Control were used.

Validation limits were used for real-time PCR assays according to the manufacturer’s instructions. Accordingly, for Septin 9 PCR the crossing point (CP) for the positive control was less than 40.5 and the negative control had no CP. For ACTB PCR, the positive control was less than 30.3 CP and the negative control was less than 37.1 CP. The PCR validity limits were different for the patient samples. In the positive cases, Septin 9 PCR CP was less than 50 and ACTB PCR CP was less than 33.7, while the Septin 9 negative cases showed no CP in Septin 9 PCR and ACTB PCR CP was less than 33.7. All of the amplification curves were regularly shaped; otherwise they were excluded as invalid measurements.

To be comparable to previous studies using SEPT9 (deVos *et al*. [16]), we first analyzed the data using a 1/3 rule in which a sample was declared positive if 1 of 3 PCR replicates had a valid curve (1/3 analysis method). Thus, a sample was considered to be positive for Septin 9 if at least one of the three Septin 9 PCRs were positive and a sample was considered to be negative for Septin 9 if all 3 of the Septin 9 PCR replicates were negative. In addition, we also analysed the data using a 2/3 rule, whereby to be called positive 2 of the 3 PCR replicates must have valid curves, following the instructions for use of the manufacturer. (2/3 analysis method). Application of this rule results in increased specificity at a lower sensitivity of detection.

### Guaiac-based Fecal Occult Test (gFOBT)

All fecal samples for gFOBT (Hema Screen, Immunostics. Inc., New Jersey) were taken at least 2 days before bowel preparation by patients. The test was done in the Central Laboratory of Semmelweis University. The detection level of stool blood was 0.6 mg Hg/gm of feces.

### Carcinoembryonic Antigen (CEA)

All blood samples for the CEA test (Cobas, Roche Diagnostics) were taken at least 2 days before bowel preparation. The test was done in the Central Laboratory of Semmelweis University. The measurement range of the *in vitro* test is 0.2–1,000 ng/mL of CEA. A CEA serum level higher than 4.3 ng/mL was considered to be in the pathological range (elevated CEA).

### Statistical Analysis

Sensitivity and specificity were calculated using a binary classification test. Sensitivity was measured as a proportion of true positive cases to the number of true positive and false negative cases. In the case of specificity, the number of true negative cases was divided by the cumulative number of all true negative and false positive results. Chi-square tests were used to estimate and test the association between healthy subjects and cancer cases and between different colon sides in tumor samples. Statistically significant (p<0.05) differences were visualized on the basis of the Pearson residuals. There results were summarized in a graphical association plot using R programming language.

## Results

### SEPT9 Sensitive Qualitative PCR

Our study included 94 healthy controls and 93 patients with colorectal cancer (57 of left-sided and 36 right-sided). Forty patient samples (16 NED and 24 CRC samples) were measured twice for validation purposes, only three of which showed contradictory results. The samples from two NED subjects and one CRC patient yielded both Septin 9 positive and negative results; hence they were excluded from the study. Therefore, 92 NED and 92 CRC (56 left-sided and 36 right-sided) were ultimately included in the analyses.

**Figure 2:**
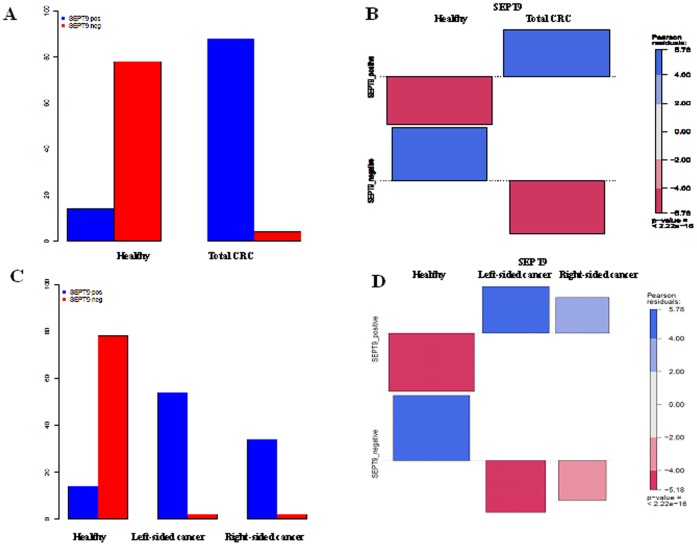
Methylated Septin 9 in healthy subjects and colorectal cancer (left-and right-sided) cases. A, B: Bar chart and association plot of SEPT9 methylation level in healthy and colorectal cancer cases. C, D: Bar chart and association plot of SEPT9 methylation in healthy, left-sided cancer and right-side cancer samples. CRC = colorectal cancer; SEPT9 = Septin 9.

We found Septin 9 methylation in 15.2% (14/92) of healthy controls and 95.6% (88/92) of CRC patients (Table 2) using 1/3 analysis method. Thus, the specificity and sensitivity of Septin 9 for CRC was 84.8% (95% confidence intervals 75.8% to 91.4%) and 95.6% (95% confidence intervals 89.2% to 98.8%), respectively (Table 3). CRC samples were then divided into left- and right-sided cancers, and in the course of this comparison no significant (p = 0.65) difference was found between the two groups. Septin 9 was methylated in 96.4% (54/56) of left-sided CRC and 94.4% (34/36) of right-sided CRC. Only 4/92 (4.3%) of CRC cases were Septin 9 negative, 2 (2/56, 3.6%) from the left-side and 2 (2/36, 5.5%) from the right side (Table 2). All CRC cases of clinical stage II or greater were detected by Septin 9 (Table 4, Figure 2).

**Table 2:** SEPT9, gFOBT and CEA results from healthy subjects and cancer patients.

	Healthy(%)	Total cancer (%)	Left-sided cancer (%)	Right-sided cancer (%)
SEPT9 positive*	14/92 (15.2)	88/92 (95.6)	54/56 (96.4)	34/36 (94.4)
SEPT9 negative*	78/92 (84.8)	4/92 (4.3)	2/56 (3.6)	2/36 (5.5)
SEPT9 positive**	1/92 (1)	73/92 (79.3)	48/56 (85.7)	25/36 (69.4)
SEPT9 negative**	91/92 (99)	19/92 (20.6)	8/56 (14.3)	11/36 (30.6)
gFOBT positive	5/17 (29.4)	15/22 (68.2)	10/12 (83.3)	5/10 (50)
gFOBT negative	12/17 (70.6)	7/22 (31.8)	2/12 (16.7)	5/10 (50)
Elevated CEA	4/27 (14.8)	14/27 (51.8)	9/15 (60)	5/12 (41.7)
Normal CEA	23/27 (85.2)	13/27 (48.1)	6/15 (40)	7/12 (58.3)

SEPT9 = Septin 9, gFOBT = guaiac-based fecal occult blood test; CEA = carcinoembryonic antigen.

* 1/3 analysis method.

** 2/3 analysis method.

**Table 3:** Sensitivity and specificity of FOBT, CEA and SEPT9.

	Sensitivity (%)	95% CI (%)	Specificity (%)	95% CI (%)
gFOBT	68.2	45.1–86.1	70.6	44–89.7
CEA	51.8	31.9–71.3	85.2	66.3–95.8
SEPT9*	95.6	89.2–98.8	84.8	75.8–91.4
SEPT9**	79.3	69.6–87.1	98.9	94.1–100

gFOBT = guaiac-based fecal occult blood test; CEA = carcinoembryonic antigen; SEPT9 = Septin 9; CI = Confidence interval.

* 1/3 analysis method.

** 2/3 analysis method.

Using the 2/3 analysis method, we observed 99% specificity (95% C.I. 94.1–100) with 91/92 of NED samples called negative, and 79.3% sensitivity (95% C.I. 69.6–87.1) with 73/92 of the CRC samples called positive (Table 3). Using the 2/3 analysis detection of left-sided (48/56; 85.7%) and right-sided (25/36;69.4%) tumors showed some difference. However because of the lower sensitivity, stage I CRC cases showed only 60% positivity and from stage II until stage IV we found a decreasing tendency of Septin 9 methylation (Table 2, 4).

**Table 4:** SEPT9 results in cancer patients according to stages.

	Total CRC (%)	Stage I(%)	Stage II(%)	Stage III (%)	Stage IV (%)
SEPT9 positive*	88/92 (95.6)	21/25 (84)	14/14 (100)	35/35 (100)	18/18 (100)
SEPT9 positive**	73/92 (79.3)	15/25 (60)	13/14 (92.8)	31/35 (88.6)	14/18 (77.8)

SEPT9 = Septin 9.

* 1/3 analysis method.

** 2/3 analysis method.

### Analysis of Guaiac-based Fecal Occult Blood Test

Occult blood was detected in the feces of 29.4% (5/17) of healthy subjects and 68.2% (15/22) of CRC patients. Thus, the specificity and sensitivity of gFOBT for CRC was 70.6% (95% confidence intervals 44% to 89.7%) and 68.2% (95% confidence intervals 45.1% to 86.1%, respectively. A nearly significantly (p = 0.09) higher proportion of left-sided CRC (83.3%; 10/12) showed gFOBT positivity compared to right-sided CRC (50%; 5/10) (Table 2, 3).

### Analysis of Carcinoembryonic Antigen Serum Level

In the case of CEA, two groups were defined: one with elevated CEA serum levels and the other with normal levels. In our study, 14.8% (4/27) of the healthy subjects had elevated CEA levels, while only 51.8% (14/27) of the CRC specimens showed elevated levels. Thus, the specificity and sensitivity of CEA for CRC was 85.2% (95% confidence intervals 66.3% to 95.8%) and 51.8% (95% confidence intervals 31.9% to 71.3%), respectively. There was no significant difference (p = 0.34) between the proportion of left-sided CRC cases (9/15, 60%) and right-sided CRC cases (5/12, 41.6%) with elevated CEA serum levels (Tables 2, 3).

## Discussion

CRC screening to identify tumors at early stages reduces the mortality of the disease. However, the highly sensitive and specific screening methods (i.e. colonoscopy and CT enterography) are invasive and patient compliance is low. On the other hand, other methods that are not as invasive (i.e., FOBT and CEA) have low specificity and sensitivity. Therefore, a suitable screening method with minimal invasiveness is needed.

In this study, we compared the sensitivity and specificity of methylated Septin 9 as a colorectal biomarker in serum to both gFOBT and CEA serum level. We performed the analysis of gFOBT testing retrospectively for both healthy controls and CRC patients. The 68.2% sensitivity of gFOBT for CRC in our study correlates to previously published results [3] and the specificity of gFOBT for CRC reached 70.6%. While this method has been used for CRC screening for several decades, it has poor sensitivity due to its non-specificity for gastrointestinal bleeding. It has been shown to reduce both CRC incidence and mortality by 15–33%. However, testing once is not sufficient, so repeated testing is needed, and in the case of positivity, colonoscopy is recommended. Unfortunately, notable numbers of patients refuse both the repeated gFOBT and the suggested colonoscopy [21]. In our study, occult blood detection from stool was performed only once for each case and gFOBT showed 29.4% (5/17) positivity in the healthy group. However, subsequent colonoscopy did not find any sign of neoplasia or polyps in these cases. Nevertheless fecal occult bleeding can be caused by upper GI bleeding, hemorrhoids, local inflammation of the colon, or dietary failure. Correlation analysis of gFOBT and SEPT9 in the same healthy samples showed specificity of 70.6% (12/17 negative) for gFOBT and 76.5% (13/17 negative) for SEPT9. For CRC, all of the patients were SEPT9 positive (100%; 22/22) whereas only 68.2% (15/22) were positive by gFOBT.

Compared to gFOBT, we found the CEA assay was more precise for detecting CRC, with 85.2% specificity, but was also less sensitive (51.8%). CEA is the most widely used serum tumor marker for CRC monitoring. This antigen is expressed on the surface of the colonic epithelial cells and in the case of malignancy, CEA is expressed on the whole cell surface and excreted at high levels into the bloodstream [22]. Elevated preoperative CEA is associated with reduced survival after surgical resection of CRC. Monitoring of CEA in the postoperative period can help to identify patients with metastasis, although its sensitivity for pulmonary metastasis is less than for hepatic metastasis [23]. The primary use of CEA is in follow-up of CRC after surgical or chemotherapy treatment. In our study, only 51.8% (14/27) of CRC patients showed elevated CEA levels. From the comparison of CEA and Septin 9 in the same patients, the specificity of the CEA assay was 85.2% (23/27), higher than the Septin 9 specificity (70.4%; 19/27); however Septin 9 was much more sensitive (100%; 27/27) in cancer samples than CEA (51.8%; 14/27). Hence serum CEA is not as a reliable method for CRC screening as Septin 9 [24].

Septins have been originally detected in cell division cycle mutant yeast, but recently it is become known that Septin proteins play role in several cellular functions. They have been implicated in neoplasia, neurological and infectious diseases [25]. Previous studies used the first generation Septin 9 detection kit (Epi proColon 1.0) and found Septin 9 positivity in 9% of healthy subjects and 73% of CRC patients [14]–[17]. In our study, new generation Septin 9 blood test, the Epi proColon 2.0 was used. The advantages of Epi proColon 2.0 kit are fewer handling steps, shorter time to result and increased clinical performance compared to the first generation test [26]. In the present study, we first analyzed the data using the high sensitivity (1/3 analysis method) as described previously [16]. In the current study only 4 of the 92 (4.3%) CRC cases showed SEPT9 negativity, all of them stage I CRC, and the test had 95.6% sensitivity for CRC. Its specificity (84.8%) was similar to that found for CEA (Table 3). Seventy-eight of the 92 (84.8%) healthy subjects were negative for Septin 9. Healthy subjects that were positive for Septin 9 did not show any sign of colonic disease at the time of colonoscopy. However, it remains to be seen whether they will develop any illness in the future. Septin 9 was sufficiently sensitive to detect early stage CRC as well. For stage I CRC, 95.6% were identified by Septin 9 methylation, and all of the stage II CRC samples showed positive test results. In case of gFOBT and CEA, the sample number was too low, preventing any analysis by CRC stage.

Using an alternative higher specificity rule (2/3 analysis), according to the manufacturer’s suggestion, we observed a specificity of 98.9%. Only 1% (1/92) of the NED group was SEPT9 positive, so this method is more suitable to detect the NED samples correctly as healthy compared with 1/3 analysis. Applying this rule to the CRC cases, 73 samples or 79.3% showed SEPT9 positivity, a level of sensitivity that outperforms gFOBT and CEA for the detection of CRC. The selection of a higher sensitivity or higher specificity algorithm may be driven by differing objectives of screening programs.

In this study, we also compared the sensitivity of methylated Septin 9 as a serum biomarker for both left- and right-sided CRC to gFOBT and CEA serum level. Recent studies have demonstrated differences in the molecular patterns of colorectal carcinogenesis based on factors such as age, gender, and tumor localization [27]–[30]. The elevated number of diagnosed left-sided colon cancers may be due to anatomical factors such as the lower colonic lumen diameter. Ghazi *et al.* found that left-sided CRC tended to have a lower T stage and higher N stage. However, they found no significant difference in the number of involved lymph nodes between the colonic locations. In addition, right-sided CRC has a worse prognosis than left-sided [31] and Weiss *et al.* found a higher mortality rate in right-sided stage III CRC [32]. While right-sided tumors display elevated gene expression levels of cell cycle control and Wnt signaling genes, left-sided colon cancers show reduced expression of tumor suppressor genes and cytokeratin 20 and elevated expression of COX-1 and genes that promote stromal expansion [33], [34]. Furthermore, difference in tumorigenesis between left- and right-sided colon cancers may be caused by epigenetic factors, as shown by differences in methylation. In regard to DNA methylation, it is known that some right-sided (CIMP) colon cancers have more frequent alterations as compared to left-sided cancers. Left-sided colon cancers can display a mutator phenotype, while right-sided tumors display as hypermethylator phenotype [35]–[37].

Taken together, it is of significant clinical importance that screening methods for CRC are reliable for both left- and right-sided CRC.

CRC detection by gFOBT in the context of left and right side of the colon has been evaluated in previous studies. Steele *et al.* found gFOBT to be less sensitive for both rectal cancer and right-sided cancer of the colon [38]. In our study, more left-sided CRC (83%) cases were detected by gFOBT than right-sided CRCs (50%) as well. The reason for detecting more left sided CRC may be explained by the localization of the disease and, due to stool consistency, blood originating from left-sided cancers may appear earlier in the feces. CEA showed less of a difference in detection between left- (60%) and right-sided tumors (42%) than gFOBT. We did not detect any difference in Septin 9 positivity between left- (96%) and right-sided CRC (94%), so the sensitivity for detecting cancer was independent of the location of the tumor. In addition, Septin 9 was by far the most sensitive marker for detecting right-sided tumors.

In conclusion, while CRC screening can potentially reduce mortality from colorectal cancer, the current CRC screening tests have unsatisfactory sensitivity and specificity or are highly invasive. Since left-sided CRC is more commonly detected than right-sided CRC by both colonoscopy and blood detection in stool, these screening methods are associated with reduced mortality from CRC arising in the left side of the colon but not from the right side [39], [40]. Hence, both improving the compliance of patients and developing more sensitive methods for right-sided CRC are needed. Peripheral blood based methods may raise patient compliance. Our report assesses the possible differences of blood-based SEPT9, gFOBT, and CEA between left- and right-sided CRCs. SEPT9 is a sensitive biomarker for the detection of CRC with the sensitivity of 100% for stage II–IV CRC. This marker was more sensitive and specific than gFOBT and CEA and did not show any differences between left- and right-sided colon cancers. Hence, the Septin 9 marker may be a safe and useful test for CRC screening.

## Supporting Information

Table S1 Individual results of gFOBT, CEA and SEPT9 for all patinets; NED = no evidence of disease; CRC = colorectl cancer; FOBT = fecal occult blood test; CEA = carcinoembryonic antigen; SEPT9 = Septin 9.
(DOC)
