# Supplementary material for: Detection of Methylated SEPT9 in Plasma Is a Reliable Screening Method for Both Left- and Right-Sided Colon Cancers
Source: PLoS One. 2012 Sep 25;7(9):e46000. doi: 10.1371/journal.pone.0046000 (PMC3457959; doi:10.1371/journal.pone.0046000)
Supplement: Table S1 — Individual results of gFOBT, CEA and SEPT9 for all patinets; NED = no evidence of disease; CRC = colorectl cancer; FOBT = fecal occult blood test; CEA = carcinoembryonic antigen; SEPT9 = Septin 9. (DOC) [file pone.0046000.s001.doc]

| Patients nr. | Diagnosis | SEPT9 | SEPT9 PCR positivity | Gender (1=male, 2=female) | Age | FOBT | CEA | Side | Stage |  |
| --- | --- | --- | --- | --- | --- | --- | --- | --- | --- | --- |
| 1 | NED | neg | 0/0 | 1 | 68 | - | norm | - | - |  |
| 2 | NED | neg | 0/0 | 2 | 65 | - | norm | - | - |  |
| 3 | NED | neg | 0/0 | 2 | 56 | neg | - | - | - |  |
| 4 | NED | neg | 0/0 | 1 | 63 | - | - | - | - |  |
| 5 | NED | neg | 0/0 | 2 | 76 | - | - | - | - |  |
| 6 | NED | neg | 0/0 | 2 | 61 | - | norm | - | - |  |
| 7 | NED | neg | 0 | 2 | 72 | - | norm | - | - |  |
| 8 | NED | neg | 0 | 2 | 53 | - | - | - | - |  |
| 9 | NED | neg | 0 | 1 | 62 | pos | norm | - | - |  |
| 10 | NED | neg | 0 | 2 | 69 | - | norm | - | - |  |
| 11 | NED | neg | 0 | 1 | 53 | - | norm | - | - |  |
| 12 | NED | neg | 0 | 2 | 62 | - | - | - | - |  |
| 13 | NED | neg | 0 | 2 | 69 | - | - | - | - |  |
| 14 | NED | pos | 1 | 2 | 62 | - | - | - | - |  |
| 15 | NED | neg | 0/0 | 2 | 63 | - | - | - | - |  |
| 16 | NED | pos | 1 | 1 | 67 | - | norm | - | - |  |
| 17 | NED | neg | 0 | 1 | 66 | - | - | - | - |  |
| 18 | NED | neg | 0/0 | 1 | 74 | neg | - | - | - |  |
| 19 | NED | neg | 0/0 | 1 | 53 | - | high | - | - |  |
| 20 | NED | pos | 1 | 2 | 68 | - | - | - | - |  |
| 21 | NED | neg | 0 | 1 | 57 | - | - | - | - |  |
| 22 | NED | neg | 0 | 1 | 69 | - | - | - | - |  |
| 23 | NED | neg | 0 | 2 | 55 | - | - | - | - |  |
| 24 | NED | neg | 0 | 1 | 52 | - | - | - | - |  |
| 25 | NED | pos | 1 | 2 | 57 | - | - | - | - |  |
| 26 | NED | neg/pos | 0/1 | 2 | 64 | - | - | - | - | excluded |
| 27 | NED | neg/pos | 0/1 | 2 | 59 | neg | norm | - | - | excluded |
| 28 | NED | neg | 0 | 2 | 80 | - | norm | - | - |  |
| 29 | NED | neg | 0 | 1 | 61 | - | - | - | - |  |
| 30 | NED | neg | 0/0 | 2 | 61 | - | - | - | - |  |
| 31 | NED | pos | 1/1 | 1 | 66 | - | - | - | - |  |
| 32 | NED | neg | 0/0 | 1 | 62 | - | - | - | - |  |
| 33 | NED | neg | 0 | 2 | 74 | - | - | - | - |  |
| 34 | NED | neg | 0 | 2 | 57 | - | - | - | - |  |
| 35 | NED | neg | 0 | 2 | 53 | - | - | - | - |  |
| 36 | NED | pos | 1 | 2 | 67 | - | - | - | - |  |
| 37 | NED | neg | 0 | 2 | 54 | - | - | - | - |  |
| 38 | NED | neg | 0 | 2 | 71 | - | - | - | - |  |
| 39 | NED | neg | 0/0 | 2 | 63 | - | norm | - | - |  |
| 40 | NED | neg | 0/0 | 1 | 55 | neg | - | - | - |  |
| 41 | NED | neg | 0 | 2 | 77 | - | - | - | - |  |
| 42 | NED | neg | 0 | 2 | 67 | - | - | - | - |  |
| 43 | NED | neg | 0 | 1 | 40 | neg | - | - | - |  |
| 44 | NED | pos | 1 | 1 | 70 | neg | norm | - | - |  |
| 45 | NED | neg | 0 | 1 | 58 | - | - | - | - |  |
| 46 | NED | neg | 0 | 2 | 78 | - | - | - | - |  |
| 47 | NED | pos | 1 | 2 | 51 | neg | norm | - | - |  |
| 48 | NED | neg | 0 | 2 | 50 | - | - | - | - |  |
| 49 | NED | pos | 2 | 1 | 80 | - | norm | - | - |  |
| 50 | NED | neg | 0 | 2 | 79 | pos | norm | - | - |  |
| 51 | NED | neg | 0 | 2 | 78 | neg | - | - | - |  |
| 52 | NED | pos | 1 | 1 | 72 | pos | norm | - | - |  |
| 53 | NED | neg | 0 | 1 | 75 | - | high | - | - |  |
| 54 | NED | neg | 0 | 2 | 70 | - | - | - | - |  |
| 55 | NED | neg | 0 | 2 | 59 | - | - | - | - |  |
| 56 | NED | neg | 0 | 1 | 53 | - | - | - | - |  |
| 57 | NED | neg | 0 | 1 | 55 | - | - | - | - |  |
| 58 | NED | neg | 0 | 2 | 66 | - | - | - | - |  |
| 59 | NED | neg | 0 | 2 | 65 | - | - | - | - |  |
| 60 | NED | neg | 0 | 2 | 62 | neg | norm | - | - |  |
| 61 | NED | neg | 0 | 2 | 24 | - | norm | - | - |  |
| 62 | NED | neg | 0 | 2 | 28 | - | - | - | - |  |
| 63 | NED | neg | 0 | 2 | 66 | neg | - | - | - |  |
| 64 | NED | pos | 1 | 1 | 68 | neg | high | - | - |  |
| 65 | NED | neg | 0 | 1 | 60 | - | - | - | - |  |
| 66 | NED | neg | 0 | 1 | 71 | - | - | - | - |  |
| 67 | NED | neg | 0 | 2 | 63 | - | - | - | - |  |
| 68 | NED | neg | 0 | 2 | 55 | - | - | - | - |  |
| 69 | NED | neg | 0 | 1 | 55 | - | - | - | - |  |
| 70 | NED | pos | 1 | 1 | 60 | - | norm | - | - |  |
| 71 | NED | neg | 0 | 2 | 59 | neg | norm | - | - |  |
| 72 | NED | neg | 0 | 2 | 51 | pos | - | - | - |  |
| 73 | NED | neg | 0 | 1 | 54 | neg | - | - | - |  |
| 74 | NED | neg | 0 | 1 | 68 | - | - | - | - |  |
| 75 | NED | neg | 0 | 2 | 60 | - | - | - | - |  |
| 76 | NED | neg | 0 | 1 | 66 | - | - | - | - |  |
| 77 | NED | neg | 0 | 1 | 81 | - | - | - | - |  |
| 78 | NED | neg | 0 | 2 | 62 | - | norm | - | - |  |
| 79 | NED | pos | 1 | 2 | 73 | - | norm | - | - |  |
| 80 | NED | neg | 0 | 2 | 63 | - | - | - | - |  |
| 81 | NED | pos | 1 | 2 | 76 | - | - | - | - |  |
| 82 | NED | neg | 0 | 1 | 63 | - | - | - | - |  |
| 83 | NED | neg | 0 | 2 | 60 | - | - | - | - |  |
| 84 | NED | neg | 0 | 1 | 76 | - | - | - | - |  |
| 85 | NED | neg | 0 | 2 | 66 | - | norm | - | - |  |
| 86 | NED | neg | 0 | 2 | 43 | - | - | - | - |  |
| 87 | NED | neg | 0 | 2 | 54 | - | - | - | - |  |
| 88 | NED | neg | 0 | 2 | 62 | - | - | - | - |  |
| 89 | NED | neg | 0 | 1 | 61 | - | norm | - | - |  |
| 90 | NED | neg | 0 | 2 | 61 | - | high | - | - |  |
| 91 | NED | neg | 0 | 2 | 57 | - | - | - | - |  |
| 92 | NED | neg | 0 | 2 | 55 | - | - | - | - |  |
| 93 | NED | neg | 0 | 2 | 65 | pos | - | - | - |  |
| 94 | NED | neg | 0 | 1 | 66 | - | - | - | - |  |
| 95 | CRC | pos | 2/3 | 2 | 68 | - | - | L | I |  |
| 96 | CRC | pos | 3/3 | 2 | 70 | pos | - | R | I |  |
| 97 | CRC | neg | 0 | 1 | 79 | - | - | R | I |  |
| 98 | CRC | pos | 1 | 1 | 75 | - | - | R | I |  |
| 99 | CRC | pos | 3/3 | 2 | 68 | - | - | L | I |  |
| 100 | CRC | pos | 3/3 | 2 | 73 | - | - | L | I |  |
| 101 | CRC | pos | 3/3 | 2 | 56 | - | high | L | I |  |
| 102 | CRC | pos | 1/3 | 2 | 74 | pos | - | R | I |  |
| 103 | CRC | pos | 3 | 1 | 62 | - | norm | L | I |  |
| 104 | CRC | pos | 3 | 1 | 77 | - | - | L | I |  |
| 105 | CRC | pos | 3/3 | 1 | 59 | - | - | L | I |  |
| 106 | CRC | pos | 1/1 | 2 | 78 | - | - | R | I |  |
| 107 | CRC | pos | 3/3 | 1 | 61 | - | - | L | I |  |
| 108 | CRC | pos | 3 | 1 | 62 | - | high | L | I |  |
| 109 | CRC | pos | 3 | 1 | 61 | - | - | L | I |  |
| 110 | CRC | pos | 1 | 1 | 64 | - | - | R | I |  |
| 111 | CRC | pos | 1 | 1 | 87 | - | - | L | I |  |
| 112 | CRC | neg | 0 | 1 | 73 | - | - | R | I |  |
| 113 | CRC | pos | 2 | 1 | 66 | - | - | R | I |  |
| 114 | CRC | pos | 1 | 2 | 54 | pos | norm | R | I |  |
| 115 | CRC | pos | 2 | 1 | 59 | - | - | L | I |  |
| 116 | CRC | pos | 3 | 2 | 55 | - | - | L | I |  |
| 117 | CRC | neg | 0 | 2 | 52 | - | - | L | I |  |
| 118 | CRC | neg | 0 | 1 | 65 | - | - | L | I |  |
| 119 | CRC | pos | 1 | 1 | 50 | - | - | L | I |  |
| 120 | CRC | pos | 3 | 2 | 78 | - | - | L | II |  |
| 121 | CRC | pos | 3/3 | 1 | 74 | pos | high | L | II |  |
| 122 | CRC | pos | 3 | 1 | 54 | - | - | L | II |  |
| 123 | CRC | pos | 3 | 2 | 74 | - | - | R | II |  |
| 124 | CRC | pos | 3 | 1 | 65 | - | - | L | II |  |
| 125 | CRC | pos | 1 | 2 | 67 | - | norm | L | II |  |
| 126 | CRC | pos | 3 | 1 | 67 | - | norm | R | II |  |
| 127 | CRC | pos | 3 | 2 | 56 | - | - | L | II |  |
| 128 | CRC | pos | 3 | 1 | 73 | - | - | R | II |  |
| 129 | CRC | pos | 3 | 2 | 57 | - | - | L | II |  |
| 130 | CRC | pos | 3 | 1 | 69 | - | - | R | II |  |
| 131 | CRC | pos | 2 | 1 | 67 | - | - | R | II |  |
| 132 | CRC | pos | 3 | 2 | 66 | - | - | L | II |  |
| 133 | CRC | pos | 2 | 2 | 62 | - | - | R | II |  |
| 134 | CRC | pos | 3/3 | 1 | 58 | - | norm | R | III |  |
| 135 | CRC | pos | 3 | 1 | 65 | - | - | L | III |  |
| 136 | CRC | pos | 3 | 1 | 72 | - | high | L | III |  |
| 137 | CRC | pos | 2/3 | 1 | 68 | - | - | L | III |  |
| 138 | CRC | pos | 3 | 2 | 50 | pos | high | L | III |  |
| 139 | CRC | pos | 2 | 2 | 88 | - | - | R | III |  |
| 140 | CRC | pos/neg | 0/2 | 2 | 81 | - | - | L | III | excluded |
| 141 | CRC | pos | 3 | 1 | 53 | - | - | R | III |  |
| 142 | CRC | pos | 1 | 1 | 50 | - | - | R | III |  |
| 143 | CRC | pos | 3 | 2 | 81 | - | - | L | III |  |
| 144 | CRC | pos | 3/3 | 1 | 68 | pos | high | L | III |  |
| 145 | CRC | pos | 3/3 | 2 | 71 | - | - | L | III |  |
| 146 | CRC | pos | 3/3 | 1 | 79 | - | - | L | III |  |
| 147 | CRC | pos | 3 | 2 | 71 | - | - | L | III |  |
| 148 | CRC | pos | 2/2 | 2 | 69 | pos | - | L | III |  |
| 149 | CRC | pos | 2 | 1 | 65 | pos | - | L | III |  |
| 150 | CRC | pos | 1/1 | 2 | 67 | pos | norm | R | III |  |
| 151 | CRC | pos | 3 | 1 | 70 | pos | - | R | III |  |
| 152 | CRC | pos | 3 | 2 | 62 | neg | norm | L | III |  |
| 153 | CRC | pos | 3/3 | 2 | 81 | neg | norm | L | III |  |
| 154 | CRC | pos | 2/3 | 2 | 76 | - | - | R | III |  |
| 155 | CRC | pos | 3 | 1 | 75 | - | - | L | III |  |
| 156 | CRC | pos | 3/3 | 1 | 83 | pos | high | L | III |  |
| 157 | CRC | pos | 3/3 | 1 | 65 | - | - | R | III |  |
| 158 | CRC | pos | 3/3 | 1 | 66 | - | - | L | III |  |
| 159 | CRC | pos | 3 | 2 | 54 | - | norm | L | III |  |
| 160 | CRC | pos | 3 | 2 | 67 | - | norm | R | III |  |
| 161 | CRC | pos | 3 | 2 | 90 | - | norm | R | III |  |
| 162 | CRC | pos | 3 | 2 | 79 | - | - | L | III |  |
| 163 | CRC | pos | 3 | 2 | 68 | pos | - | L | III |  |
| 164 | CRC | pos | 3 | 1 | 57 | pos | norm | L | III |  |
| 165 | CRC | pos | 1 | 1 | 66 | - | - | L | III |  |
| 166 | CRC | pos | 3 | 2 | 67 | - | - | R | III |  |
| 167 | CRC | pos | 3 | 2 | 82 | - | - | L | III |  |
| 168 | CRC | pos | 1 | 1 | 70 | - | - | L | III |  |
| 169 | CRC | pos | 3 | 2 | 66 | - | - | R | III |  |
| 170 | CRC | pos | 3 | 1 | 70 | - | - | L | IV |  |
| 171 | CRC | pos | 3 | 2 | 76 | - | - | R | IV |  |
| 172 | CRC | pos | 1 | 1 | 64 | - | - | L | IV |  |
| 173 | CRC | pos | 3/3 | 1 | 60 | - | high | R | IV |  |
| 174 | CRC | pos | 3 | 2 | 50 | - | - | R | IV |  |
| 175 | CRC | pos | 3 | 2 | 70 | pos | high | L | IV |  |
| 176 | CRC | pos | 3 | 2 | 74 | neg | high | R | IV |  |
| 177 | CRC | pos | 3 | 1 | 69 | - | - | L | IV |  |
| 178 | CRC | pos | 3 | 1 | 87 | - | - | L | IV |  |
| 179 | CRC | pos | 1 | 2 | 91 | neg | norm | R | IV |  |
| 180 | CRC | pos | 3 | 1 | 67 | neg | high | R | IV |  |
| 181 | CRC | pos | 1 | 2 | 59 | neg | high | R | IV |  |
| 182 | CRC | pos | 3 | 1 | 46 | - | - | L | IV |  |
| 183 | CRC | pos | 3 | 2 | 57 | pos | high | L | IV |  |
| 184 | CRC | pos | 3 | 2 | 69 | neg | high | R | IV |  |
| 185 | CRC | pos | 3 | 1 | 65 | - | - | L | IV |  |
| 186 | CRC | pos | 1 | 2 | 70 | - | - | R | IV |  |
| 187 | CRC | pos | 3 | 1 | 85 | - | - | L | IV |  |
